# Urokinase Plasminogen Activation System Modulation in Transformed Cell Lines

**DOI:** 10.3390/ijms26020675

**Published:** 2025-01-15

**Authors:** Diana Culej Bošnjak, Tihana Balent, Petra Korać, Mariastefania Antica, Maja Matulić

**Affiliations:** 1 Department of Biology, Faculty of Science, University of Zagreb, Horvatovac 102, 10000 Zagreb, Croatia; tihanabalent@gmail.com (T.B.); petra.korac@biol.pmf.hr (P.K.); mmatulic@biol.pmf.hr (M.M.); 2 Ruđer Bošković Institute, Bijenička 54, 10000 Zagreb, Croatia; antica@irb.hr

**Keywords:** urokinase plasminogen activator, plasminogen activator inhibitor, proliferation, migration, adhesion

## Abstract

The role of the plasminogen activation system is to regulate the activity of the extracellular protease plasmin. It comprises the urokinase plasminogen activator (uPA), a specific extracellular protease which activates plasminogen, its inhibitor PAI1, and the urokinase plasminogen activator receptor, uPAR, which localizes the urokinase activity. The plasminogen activation system is involved in tissue remodeling through extracellular matrix degradation, and therefore participates in numerous physiological and pathological processes, which make it a potential biomarker. To investigate the role of these molecules in the cellular processes, we cloned human uPA, PAI1, and uPAR and overexpressed them in two cell lines, the glioblastoma line A1235 and the transformed human embryonal kidney cells HEK 293. We analyzed the urokinase activity and the expression of plasminogen activation system elements on the protein and RNA level by Western blot analysis and RTqPCR. Cell proliferation was followed up by cell counting, cell migration and invasion by wound-healing and the transwell assays, respectively, and cell adhesion and dispersal by spheroid formation. The cells transfected with urokinase sequence had increased urokinase activity and uPA expression, while the PAI1-transfected cells decreased urokinase activity, increased PAI1 expression, and decreased cell migration. HEK 293 cells expressing PAI formed only small spheroids. The effects of the uPA system molecules depended on their interactions with each other and with other molecules in the microenvironment, as well as on the cell-type-specific signaling.

## 1. Introduction

The plasminogen activation system is a complex system aiming to activate extracellular, ubiquitous proenzyme plasminogen and produce protease plasmin with a wide spectrum of substrates. These reactions are regulated through a controlled production of the urokinase plasminogen activator, an extracellular serine protease specifically targeting plasminogen. Urokinase is regulated mainly at the level of transcription [1,2]. It is secreted as a proenzyme and is activated by extracellular cleavage. In addition, its activity can be modulated by binding to its receptor, urokinase plasminogen activator receptor, uPAR, and inactivated by binding plasminogen activator inhibitor, PAI1. This system regulates extracellular matrix (ECM) degradation, present in numerous physiological and pathological processes, mainly through plasmin activity and subsequent metalloproteinase activation. Therefore, it has a role in tissue remodeling in adult organisms and during embryonal development, wound healing, and fibrin clot degradation, but also in cell migration and tumor cell invasion [1,2]. Further, the extracellular proteases can activate and influence several signaling networks through the release of small molecules and activation of growth factors, acting in the angiogenesis regulation and in the cell proliferation [3,4,5]. uPA was also found to have a role in signaling, in the synaptic plasticity in the brain and in the regulation of cell proliferation [6,7,8,9]. The whole system is often correlated to the epithelial–mesenchymal transition, and thus to the metastasis process in tumorigenesis, but the effects and the activities of the system are often cell-type-specific and the roles of uPA and PAI1 in tumorigenesis are still controversial [10,11]. To learn more about the specific roles of each of the molecules of the urokinase plasminogen activation system, we modulated the expression of uPA, PAI1 and uPAR in two tumor cell lines by overexpression of uPA system molecules, cloned in plasmids and transfected into cells. One of these cell lines has high uPA activity, while the other does not have significant expression of uPA system molecules. The cell strains overexpressing uPA, PAI1 and uPAR were tested for their proliferation, migration and adhesion ability.

## 2. Results

### 2.1. Cloning of Urokinase, PAI1 and uPAR Sequences and Production of Strains with Different Urokinase Activity

**Figure 1:**
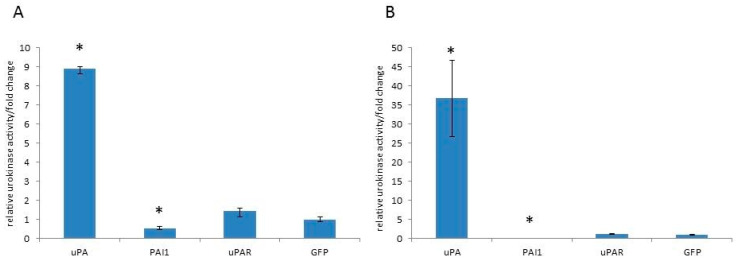
Urokinase activity of A1235 (**A**) and HEK293 (**B**) cell strains transfected with plasmids coding for the urokinase plasminogen activator system molecules. The cell strains were obtained by transfection of plasmids coding for urokinase (uPA), PAI1, uPAR and control plasmid pEGFP (GFP). The urokinase activity was determined by caseinolysis of cells’ conditioned media and by comparison with the urokinase calibration curve. The values are presented as fold changes in comparison with the values of control cells (GFP). The experiments were carried out in duplicate. uPA, PAI1, uPAR, GFP: cell strains transfected with the plasmids coding for urokinase (uPA), PAI1, uPAR and GFP, respectively. * The mean values were significantly different from the control (*p* < 0.05).

In order to investigate the role of the uPA system molecules in cell biology, we cloned human uPA, PAI1 and uPAR sequences in expression plasmids. The recombinant plasmids were assayed for insert orientation and accuracy by sequencing. A1235 glioblastoma and HEK 293 cells were transfected with recombinant plasmids, as well as with pEGFP, coding for green fluorescent protein, as a transfection control. The transfected cells were selected in geneticin for two weeks. Pooled colonies were tested for urokinase activity by caseinolysis. A1235 cells have relatively high basal urokinase activity, and the clones transfected with plasmid containing the uPA sequence showed an increase in activity of up to 9-fold (*p* < 0.05) (Figure 1A). HEK 293 cells have low basal activity, and plasmid transfection increased the activity up to 30-fold (*p* < 0.05), as presented on Figure 1B. The cells transfected with plasmids containing the PAI1 sequence significantly decreased uPA activity in both cell lines. The cells transfected with plasmid containing the receptor sequence did not cause changes in the urokinase activity when compared with the control strain transfected with pEGFP plasmid only. The cell morphology of both transfected cell lines with different plasmids was not significantly changed.

### 2.2. Expression of uPA System Molecules in Two Cell Lines

**Figure 2:**
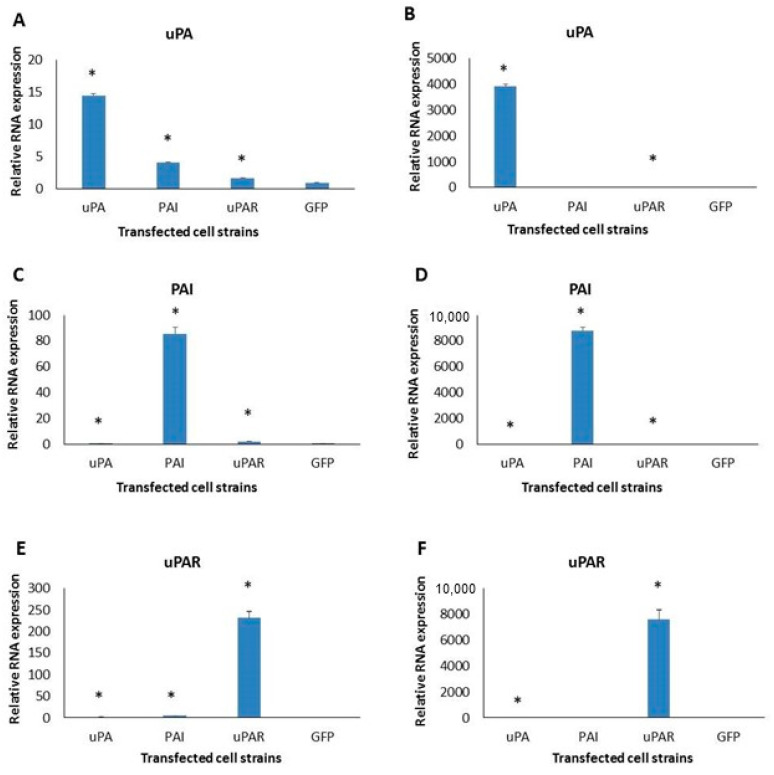
qRT-PCR analysis of urokinase plasminogen system gene expression in A1235 (**A**,**C**,**E**) and HEK293 (**B**,**D**,**F**) cell strains. The cells were transfected with the plasmids coding for urokinase (uPA), PAI1 (PAI) uPAR and pEGFP (GFP). After RNA isolation and reverse transcription, cDNA was analyzed by qRT-PCR using Syber green technique and specific primers for uPA (**A**,**B**), PAI1 (**C**,**D**), and uPAR (**E**,**F**). Gene RNA expression was normalized with HPRT expression and presented as a fold change in comparison with the expression of the GFP clones. * The mean values were significantly different from the control (*p* < 0.05).

To show that changes in the urokinase activity stem from proteins expressed from the transfected plasmids, we analyzed the expression of uPA, PAI1 and uPAR in the obtained strains. After RNA isolation and cDNA synthesis, qPCR was performed. Figure 2 shows the expression of uPA system molecules: in A1235 cells, uPA clones had 14-fold increased expression, PAI clones’ was around 80-fold and uPAR’s was more than 200-fold. A1235 cells transfected with different sequences of the uPA system molecules also changed the expression of other system members. In the uPA-transfected clones, PAI1 expression was increased 4 times, and that of uPAR was increased around 1.7 times. In PAI1-transfected clones, uPAR expression was increased 2.4 times, and in uPAR-transfected clones, PAI1 expression was increased 4 times. uPA expression was also modulated but to a lesser extent (Figure 2A,C,E). In HEK 293 clones, uPA, PAI1 and uPAR showed very high relative expression in corresponding clones of several thousand times (Figure 2B). uPA and uPAR expression was downregulated by the overexpression of different uPA system molecules in HEK 293 cells, but the basal expression of these molecules was also low.

**Figure 3:**
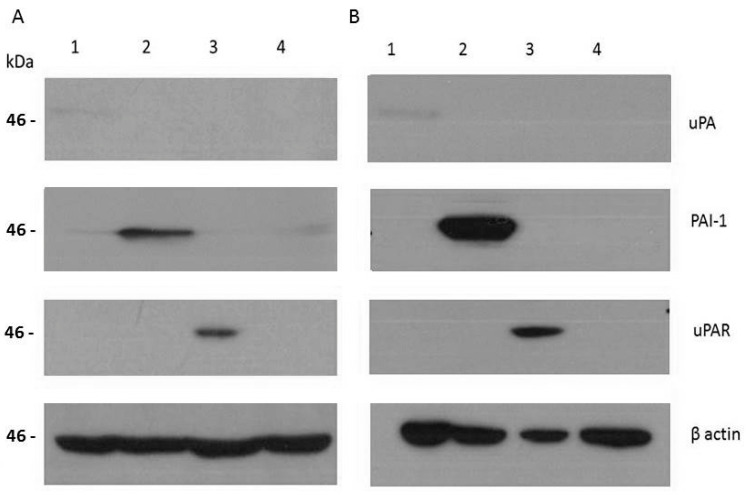
Expression of proteins in A1235 (**A**) and HEK293 (**B**) cell strains transfected with plasmids coding for urokinase plasminogen activation system. The cell lysates were prepared from the cells transfected with the plasmids coding for uPA (1), PAI1 (2), uPAR (3) and control plasmid pEGFP (4). After electrophoresis and transfer on the membrane, the proteins were incubated with the antibodies against urokinase (uPA), PAI1, uPAR and β actin, and detected by chemoluminiscence. The molecular weight of urokinase is ~50 kDa, that of PAI1 is 42–45 kDa, and that of uPAR is 35–65 kDa, dependent on glycosylation.

**Figure 4:**
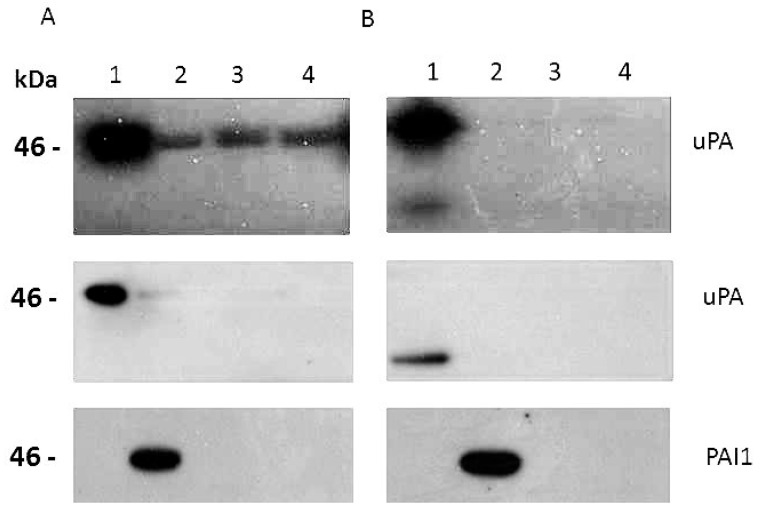
Urokinase zymography and Western blot analysis of conditioned media from the cell strains of A1235 (**A**) and HEK 293 (**B**) transfected with different plasmids coding for the plasminogen activation system molecules. Upper part: zymography made by gel overlaid on the agarose plate containing plasminogen and casein. Lower part: conditioned media samples electrophoresed transferred on the membrane further incubated with antibodies against urokinase (uPA) and PAI1. The concentrated conditioned media were from cells transfected with plasmids coding for urokinase (1), PAI1 (2), uPAR (3) and GFP (4). The molecular weights of extracellular urokinase are 50 and 30 kDa.

From the recombinant strains of A1235 and HEK 293 cells, the total proteins were isolated to analyze the expression of the uPA system molecules at the protein level. The Western blot analysis showed an increased amount of uPA, PAI1 and uPAR in the clones transfected with the corresponding plasmids (Figure 3). The molecular weights of overexpressed proteins corresponded to the wild-type proteins: urokinase has around 50 kDa, PAI1 42–45 kDa, depending on the alternative splice variants and glycosylation, and uPAR between 35 and 65 kDa, also as a consequence of cell-type-specific glycosylation [1,2]. In A1235 strains, uPA protein expression was increased 4 times, that of PAI1 16 times, and that of uPAR 47 times. A basal level of PAI1 expression in all transfected cell strains was also detected. In HEK 293 cells, uPA expression was increased 2.5 times, that of PAI1 172 times, and that of uPAR 117 times. We also concentrated conditioned media in order to detect extracellular proteins. They were analyzed by zymography for uPA activity, which was increased in A1235 and HEK 293 clones transfected with uPA sequence. Also, uPA and PAI1 were detected by Western blot in the conditioned media in the corresponding clones (Figure 4). Detected uPA was in the form of 50 kDa protein in A1235 and cleaved 30 kDa protein in HEK 293. Both are extracellular active forms [1].

### 2.3. Analysis of Cell Proliferation and Migration

**Figure 5:**
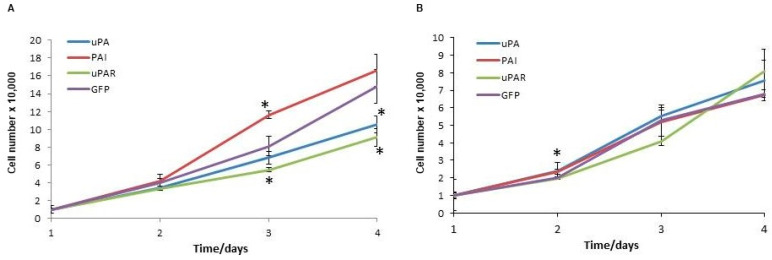
Growth curves of A1235 (**A**) and HEK 293 (**B**) cells transfected with the plasmids coding for urokinase (uPA), PAI1 (PAI), uPAR and GFP. The cells were seeded in triplicate and counted on the hemocytometer every 24 h for 4 days. * The mean values were significantly different from the control (*p* < 0.05).

To investigate the effects of the uPA system molecules on cell proliferation, we followed the cell growth of strains overexpressing uPA, PAI1 and uPAR for four days. Among the A1235 strains, the control and PAI strains proliferated faster. On the third day, the PAI strain showed a faster growth rate than the control cells, while that of uPAR was slower. On the fourth day, the uPAR and uPA strains had a lower proliferation than the control GFP strain. HEK 293 strains proliferated at a similar rate. On the second day, there was only a small difference in the PAI1 strain growth rate in comparison with the GFP cells (Figure 5).

**Figure 6:**
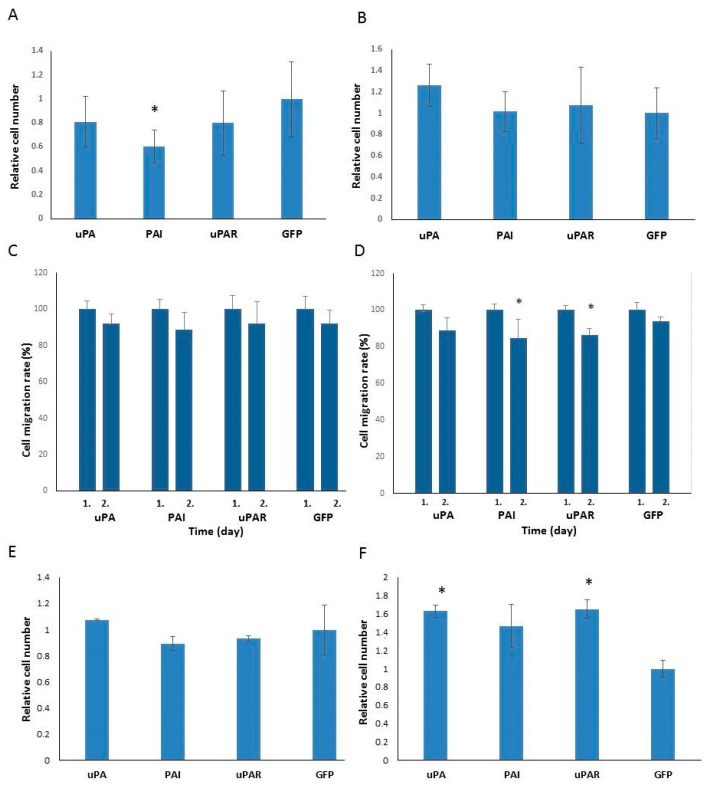
Cell migration analysis in A1235 (**A**,**C**,**E**) and HEK 293 (**B**,**D**,**F**) cell strains transfected with the plasmids coding for urokinase (uPA), PAI1 (PAI), uPAR and GFP (control cells). (**A**,**B**): Transwell migration assay: the cells were seeded in the transwell chambers in the medium without serum and allowed to migrate toward the serum supplemented medium. After 24 h, the cells that migrated through membrane pores were stained and counted by the Image J program. (**C**,**D**): Wound-healing assay: a confluent cell layer was scratched and the cells were allowed to migrate for 24 h. The dimensions of the scratch were measured at the beginning of the assay and after 24 h and compared. (**E**,**F**): Transwell invasion assay: the cells were seeded as for the migration assay, but the membrane was covered with MaxGel. (**A**,**C**,**E**): A1235 cells; (**B**,**D**,**F**): HEK 293 cells. * The mean values were significantly different from the control (*p* < 0.05).

The urokinase system is often linked to cell invasion and migration as urokinase can activate plasminogen and increase the degradation of the ECM through plasmin and by plasmin activation of metalloproteinases [12,13]. PAI1, although it decreases urokinase activity, could also be important for cell migration [14]. uPAR localizes uPA on the cell surface and has a role in uPA–PAI1 complex internalization [15]. Therefore, we analyzed the ability of cells to migrate. In a wound-healing test, comparing the dimensions of the scratched wound after 24 h, no significant differences were observed in A1235 cell strains transfected with the plasmids expressing uPA, PAI1 and uPAR in comparison with the control strain. In HEK 293 strains, a decrease in migration was observed in the cells transfected with uPAR and PAI1 (Figure 6C,D). The transwell migration test was also performed because cell proliferation can influence the results of the wound-healing assays. The number of cells migrating through the transwell membrane pores was analyzed by the ImageJ program. Among the A1235 strains, PAI1-transfected cells showed a decrease in migration, while in the HEK 293 strains, differences in migration ability were not significant (Figure 6A,B). We also analyzed the cells’ ability to invade. The cells were seeded on the transwell chambers covered with MaxGel and counted the next day. The A1235 cell strains did not show any differences in invasion ability, while the HEK 293 cells overexpressing uPA showed increased invasion. But while glioblastoma cells, in general, showed a high ability to invade, only a small number of HEK 293 cells invaded the membrane (Figure 6E,F).

### 2.4. Analysis of Cell Adhesion

**Figure 7:**
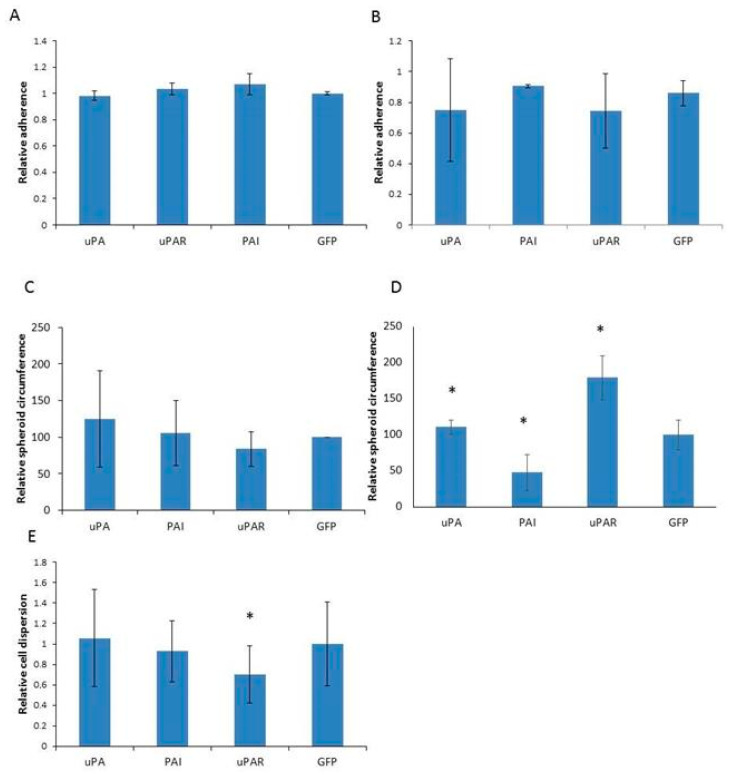
Cell adherence, spheroid formation and dispersal analysis in A1235 (**A**,**C**,**E**) and HEK 293 (**B**,**D**) cell strains transfected with plasmids coding for urokinase (uPA), PAI1 (PAI), uPAR and GFP (control cells). (**A**,**B**): Adherence analysis of cell strains expressed as the percentage of cells adherent after 30 min in comparison with the total number of adherent cells after prolonged time. (**C**,**D**): Relative circumference of spheroid 2D microphotographs after formation in hanging drops. (**E**): Relative cell dispersal as the ratio between the dispersal zone and the core spheroid size after seeding the spheroid on the adherent surface. (**A**,**C**,**E**): A1235 cells; (**B**,**D**): HEK 293 cells. * The mean values were significantly different from the control (GFP cells) (*p* < 0.05).

To investigate whether PAI1 expression influences cell dispersal and adhesion, we examined the cells’ ability to form spheroids. All cell strains, from both A1235 and HEK 293 lines, showed a high ability of adhesion, and we did not detect differences in the adhesion velocity among the cell strains (Figure 7A,B). When seeded to form spheroids, the A1235 cell strains formed spheroids of different compactness. We tried to quantify the differences in the spheroid shape by measuring the spheroid circumference on 2D microphotographs. We did not find differences in A1235 spheroid circumferences (Figure 7C). A1235 spheroids were also seeded on the adherent surface, after formation, and the dispersal zone was measured. The smallest dispersal zone was observed in uPAR-transfected cells (Figure 7E), which also formed very compact spheroids. In HEK 293 cells, one spheroid was formed in each drop by uPA, uPAR and control GFP cells, as expected, but PAI1-overexpressing cells formed many small spheroids (Figure 7D). To see whether cell density in the cell culture, from which spheroids were seeded, could influence the spheroid shape, we repeated the experiment by seeding the same number of the cells in drops from the high- and low-density cultures. The results were the same. HEK 293 spheroids, when seeded on the adherent surface, readily formed a confluent surface and could not be analyzed for dispersal.

## 3. Discussion

The urokinase plasminogen activator system has several roles in cell biology. Its functions comprise those that are connected with its proteolytic activities and involve the activation of plasminogen and extracellular matrix degradation by plasmin, but also those that are nonproteolytic. Therefore, the uPA system can be involved in cell proliferation, adhesion and migration [16]. Especially complex are processes involving cell adhesion and tumor invasion that include intricate interplay between uPAR, PAI1, uPA, and vitronectin, but also plasminogen and plasmin [16].

In order to analyze the effect of the different uPA system elements on the biology of tumor cells, we cloned and overexpressed uPA, PAI1 and uPAR in two cell lines, one known for high uPA activity and the other without significant expression of these proteins. The transfected clones overexpressed uPA, PAI1 and uPAR at the RNA and protein level, and changes in the expression of uPA and PAI1 also influenced plasmin activity. Proteolysis was increased when uPA was overexpressed and decreased in PAI1-overexpressing cells.

We observed an increase in cell proliferation in PAI1 clones, and a decrease in uPAR and uPA clones of A1235 glioblastoma cells, in comparison with GFP control cells. HEK 293 clones had in general a similar proliferation rate. The influence of uPA system elements on proliferation has been already investigated in different settings and was mainly dependent on the cell type and the expression of uPAR and the molecules cooperating with it. Most of the experiments showed that knockout of uPAR or downregulation of uPA decreased cell proliferation [17,18,19,20,21]. In some of these experiments, it was shown that processes were dependent on uPAR binding to uPA, which, in cooperation with coreceptors, could influence cell signaling. One example is cell growth regulating EGF-EGFR signaling. However, uPA–uPAR interaction was also found to activate different signaling pathways in different cell types and can also lead to inhibition of proliferation [22,23,24]. Moreover, soluble uPAR, by scavenging uPA, influenced breast cancer cell growth in vivo but not proliferation in vitro [25]. Possibly weak expression of uPAR in HEK 293 cells overexpressing uPA in our experiments influenced the absence of uPA effects on cell proliferation. Considering PAI1, it was found that its overexpression can increase proliferation, as shown in A1235 cells. Its expression was associated with the aggressiveness and the progression of bladder and gastric cancer [26,27].

As uPA and the uPA–uPAR system were shown to be involved in tumor aggressiveness, migration, metastasis and invasion in different types of tumors, such as breast, lung and ovarian, both in vitro and in vivo [19,20,21,28,29,30,31,32,33,34,35,36], we analyzed the migration of A1235 and HEK 293 cells. In A1235 cells, we observed inhibition of migration in PAI1-overexpressing cells, and in HEK 293, in PAI1- and uPAR-expressing cells. uPA over expression did not increase migration. uPA–uPAR signaling was found to be linked to epithelial–mesenchymal transition in breast cancer cells, regulated by Snail [37]. However, in some breast cancer cells, it was found that the uPA system inhibited migration, independently of its protease activity, acting as a negative modulator of EGF-dependent motility [22]. Considering PAI1 overexpression, in glioma cells, it was shown that it inhibited cell motility and invasion through ECM containing laminin and collagen, but not invasion in the 3D model of brain tissue, indicating the role of ECM composition. On the other hand, glioblastoma cell lines with knockdown of PAI1 showed decreased dispersal, and inhibited migration and movement on both uncoated and vitronectin coated surfaces [38]. In neuroblastoma, uPAR was important for the maintenance of the epithelial phenotype [11] and thus could negatively influence migration. HEK 293 also has epithelioid morphology and in uPAR-overexpressing cells, a complex process of competition and binding of uPA, PAI1 and vitronectin was described, which could influence cell migration through processes of cell adhesion to the extracellular matrix [16]. As the cells can migrate on 2D surfaces in dependence on the integrins and the adhesion to the extracellular matrix, but can also move by amoeboid type of movement through the matrix, independently on the integrins [39,40], uPAR-overexpressing HEK 293 cells change the type of cell movement but not the rate of migration [41]. Migration in these cells is independent of uPA [41].

In contrast to migration, invasion into ECM involves proteolysis and the release of extracellular proteases, such as uPA and plasmin. Originating from glioblastoma, a highly invasive tumor type, A1235 cells have an invasive ability, but did not show differences between the clones overexpressing different elements of the uPA system. Possibly, these cells use other mechanisms for cell invasion. On the contrary, in epithelial HEK 293, the number of invading cells was increased in cultures with increased uPA and uPAR expression, although these cells showed a weak invasion ability. As these cells express different types of metalloproteinases, uPA can increase their activity and influence invasion. Beside involvement in the metalloproteinase activation, uPA binding to uPAR can regulate the expression of molecules responsible for cell invasion, such as EMPPRIN (extracellular matrix metallo-proteinases inducer) and enolase [39,40]. We can assume that the effect of the uPA system on invasion depends on the cell type and its signaling, as well as on the type of ECM [38].

The processes of cell invasion and migration are tightly linked to the ECM adherence ability, especially because of the competition in binding PAI1 to uPAR and vitronectin. Among uPA-system-overexpressing glioblastoma cells, we detected decreased dispersion in uPAR-overexpressing cells. These cells also had the most compact spheroids, although not statistically significant. Among HEK 293 strains, PAI1-overexpressing cells formed only small spheroids, indicating decreased adherence in 3D system. Both cell lines express low levels of vitronectin ([42] and personal observation). PAI1 was shown to act antagonistically to cell adhesion, in dependence on its concentration. It was also shown that together with uPA, it can enhance cell adhesion to vitronectin [16]. We could assume that uPA system molecules are, in cooperation with some coreceptors, also involved in cell–cell interactions and adhesion.

Beside the effects of the uPA system on the cell microenvironment, our results also indicate that uPA system genes act in coordination and are coregulated. While the level of expression of all the uPA elements was very low in HEK 293 cells, on both the RNA and the protein level, glioblastoma cells have intrinsic expression of uPA, PAI and uPAR. In these cells, uPA overexpression increased the expression of PAI1 and uPAR. PAI1 and uPAR, when overexpressed, increased the expression of each other. uPA can act through positive feedback loops, activating the MAP kinase pathway, which regulates promoters of the uPA system genes [2,8]. PAI1 can influence signaling pathways through its complex relationship to uPAR. Moreover, in colorectal carcinoma cells, it was found that uPA and PAI1 were oppositely regulated by signaling pathways involved in EMT and wnt signaling [43].

The results of uPA system overexpression indicate that this system is involved in many biological processes which merit further investigation.

## 4. Materials and Methods

### 4.1. Cloning of uPA, PAI1 and uPAR Sequences

Human uPA, PAI1 and uPAR sequences were amplified from cDNA obtained by the total RNA reverse transcription (Primescript, Promega, Madsion, WI, USA) using oligodT primers. cDNA was obtained from A1235 glioblastoma and MDA MB231 breast cancer cell strains. The sequences were amplified using specific primers determined by IDT [44] or Primer-NCBI [45] programs and ALLin^TM^RPH Polymerase (HighQu, Kraichtal, Germany), for 35 cycles, according to the manufacturer’s protocol. The primers used for cloning are listed in Supplementary Table S1. The amplified sequences were cut out from agarose gel after electrophoresis and purified using Wizard SV Gel and PCR Clean-Up System (Promega, Madsion, WI, USA), according to the manufacturer’s instructions. PAI1 and uPAR sequences were first cloned into pGEM-T Easy vector (Promega), according to the manufacturer’s protocol, and the plasmids were used for *E. coli* XL10-Gold chemical transformation. After detection of recombinant plasmids, PAI1 and uPAR sequences were cut out from the vector using *EcoRI* restriction enzyme and recloned into the expression vector pcDNA3 (Invitrogen, Waltham, MA, USA), previously linearized with the same enzyme. After ligation and the attainment of *E. coli* colonies, the plasmids were isolated and checked for orientation by restriction analysis. The uPA sequence was ligated into pTARGET plasmid (Promega) and after *E. coli* transformation, the recombinant plasmids were isolated. The sequence orientation was determined by restriction analysis. The plasmids’ sequences were also confirmed by sequencing (Macrogen, Seoul, Republic of Korea).

### 4.2. Cell Culture, Growth Assessment and Transfection

The human glioblastoma cell line A1235 and human embryonal kidney cells HEK 293 were grown in DMEM supplemented with 10% fetal bovine serum (both from Sigma, Marlborough, MA, USA) under the standard conditions. HEK 293 cells are available at ATCC (Manassas, VA, USA), while A1235 cells were a kind gift from S. A. Aaronson (National Cancer Institute, Bethesda, MD, USA) [46]. Cell proliferation was followed up by counting cells every day on the hemocytometer under a microscope. The experiments were carried out in triplicate. For plasmid transfection, Lipofectamine 3000 Reagent (Thermo Fisher Scientific, Waltham, MA, USA) was used according to the manufacturer’s instructions. Beside recombinant plasmids with uPA, PAI1 and uPAR sequences, pEGFP plasmid (Clontech, Takara Bio, San Jose, CA, USA) coding for green fluorescence protein was used as a control. Two days after transfection, 400 µg/mL of geneticin (Calbiochem, Sigma-Aldrich, Burlington, MA, USA) was added to the medium and the cells were grown in the selection medium until the colonies were formed and collected.

### 4.3. Western Blot Analysis

Protein cell extracts were prepared in lysis buffer containing 1% nonionic detergent with the addition of protease inhibitors (Karl Roth, Karlsruhe, Germany) [47]. After electrophoresis on 10% SDS-PAGE gel, the proteins were transferred to a PVDF membrane, blocked in nonfat dried milk and the membrane probed with primary and secondary antibodies according to the standard procedure [47]. The antibodies used were specific against PAI1 (Becton Dickenson, Franklin Lakes, NJ, USA), uPA (Cell Signaling Technology, Danvers, MA, USA), uPAR (Cell Signaling Techology, Danvers, MA, USA) and β-actin (Santa Cruz, Dallas, TX, USA). The proteins were detected by chemoluminiscence on X-ray films (Biorad, Hercules, CA, USA). Densitometric analysis was performed using the ImageJ program (version 1.52a, National Institute of Health, Bethesda, MD, USA). Protein concentration was measured by the Bradford assay [47].

### 4.4. Enzyme Activity Assays

uPA activity was tested by radial caseinolysis of the conditioned media without serum on the agarose plates with plasminogen and casein as substrates [48]. The lysis zones obtained were measured and compared with a calibration curve carried out using human uPA (Leo Pharmaceutical Products, Ballerup, Denmark). The urokinase activity was normalized according to the protein concentration in lysates. Experiments were carried out in two biological replicates. For zymography, conditioned media without serum were collected from the cell cultures of different strains seeded in the same number and concentrated with Microcon 10 centrifugal filters (Merck Millipore, Louis Missouri, MO, USA) ~14-fold. Nondenatured concentrated conditioned media were run on polyacrylamide gel and, after incubation, in 2.5% Triton x-100 and water; the gel was laid on agarose gel containing casein and plasminogen and incubated on 37 °C overnight [48].

### 4.5. Migration and Invasion Assays

Cell migration was assayed by the wound-healing test and migration through transwell according to the methods reported previously [49]. The cells were seeded in DMEM without serum in a transwell chamber (Brand, Wertheim am Main, Germany) and were allowed to migrate toward DMEM supplemented with 10% bovine fetal serum overnight. The experiments were carried out in duplicate and repeated at least once. The transwell membranes were stained with crystal violet, microphotographed on the stereo microscope (Stemi 2000-C, Zeiss, Jena, Germany) and analyzed in ImageJ program (ImageJ, NIH, Bethesda, MD, USA) [50].

For the invasion assay, 5–7 × 10^4^ cells were seeded on a transwell chamber membrane coated with MaxGel ECM (Sigma-Aldrich). MaxGel was prepared according to the manufacturer’s protocol. The following procedure was the same as for cell migration.

For the scratch test, the cells were seeded in a 96-well plate and the confluent cell layer was wounded with a 100 μL volume tip. After 24 h, the dimensions of the scratch were measured. The microphotographs (Axiovert 40 CFL Zeiss with AxioCam MRm camera), taken immediately after scratching and one day after, were analyzed in the ImageJ program.

### 4.6. Spheroid Formation and Cell Adhesion Assay

The ability to form spheroids was examined according to the work of Seker et al. [38]. Briefly, 10^4^ cells in a suspension were incubated in 20 μL hanging drops on Petri dish covers, for three days, in the humid atmosphere of a CO_2_ incubator. For each cell line, around 20 drops were seeded and the experiments were repeated at least once. The formed spheres were photographed (Axiovert 40 CFL Zeiss) and measured in the ImageJ program. The cells’ dispersion was analyzed on the spheroids seeded on the multi-well plates. A day after seeding, the attached spheroids were photographed and the dispersal surface was measured and compared to the spheroid core surface in the ImageJ program [38].

To examine cell adhesion, the cells were seeded on 12-well plates and fixed with cold methanol after 30 min, 2, 3 or 4.5 h. For each time point, the cells were seeded in triplicate. The cells were stained with crystal violet, microphotographed on a stereo microscope (Stemi 2000-C, Zeiss), and analyzed in the ImageJ program.

### 4.7. qRT-PCR Assay

Total RNA was extracted from the cells using TRI Reagent (Sigma, Marlborough, MA, USA). The reverse transcription to produce cDNA was carried out with Primesript RTase (Takara, Osaka, Japan) using oligodT according to the manufacturer’s instructions. Quantitative real-time PCR (qRT-PCR) was performed using GoTaq^®^ qPCR Master Mix (Promega, Madison, WI, USA) in a 7500 Fast Real-Time PCR system (Applied Biosystems, ThermoFisher Scientific, Waltham, MA, USA). The primers for PCR reaction were published previously [18] or were designed in the programs IDT PrimerQuest software package (Integrated DNA Technologies, Inc., Coralville, IA, USA) and Primer-BLAST (NIH, Bethesda, MD, USA) (Supplementary Table S1). Gene expression was validated by comparison with HPRT gene expression and presented as a fold change in comparison with the expression in the cells transfected with control plasmid.

### 4.8. Statistical Analysis

For statistical analysis, the software package of Microsoft Office was used. The significance of independent two-tailed Student’s *t*-test was set at a *p* value < 0.05.

## 5. Conclusions

In the experiments presented, we analyzed the biological effects of the uPA system elements’ overexpression on glioblastoma and HEK 293 cells. While overexpression of uPA and uPAR led to a decrease in proliferation in the glioblastoma strains, the HEK 293 cell strains had similar proliferation rates. The clones expressing PAI1 showed decreased migration, and uPA- and uPAR-expressing HEK 293 cells had increased invasion. uPAR overexpression influenced glioblastoma cells’ dispersion, and PAI1 influenced spheroid formation in the HEK 293 cells. The effects of the uPA system molecules depend on their interactions with each other and with other molecules in the microenvironment, as well as on the cell-type-specific signaling, making this system a promising but also cell-type-specific potential target for a tumor therapy approach.

## Data Availability

The data presented in this study are available upon request from the corresponding author.

## Supplementary Materials

The following supporting information can be downloaded at: https://www.mdpi.com/article/10.3390/ijms26020675/s1.
