# Supplementary material for: Urokinase Plasminogen Activation System Modulation in Transformed Cell Lines
Source: Int J Mol Sci. 2025 Jan 15;26(2):675. doi: 10.3390/ijms26020675 (PMC11765620; doi:10.3390/ijms26020675)
Supplement: Supplementary file 1 [file ijms-26-00675-s001.zip › ijms-3345879-supplementary.pdf]

Supplement Table 1. List of primers used for PCR amplification

uPA-F (for cloning)	5'-CCCGACCTCGCCACCATGAGAGCCCTG-3'
uPA-R (for cloning)	5'-GACCCTCAGAGGGCCAGGCCATTC-3'
uPAR-F (for cloning)	5'-GGAGCTGCCCTCGCGACATGGGT-3'
uPAR-R (for cloning)	5'-TCAGGTTTAGGTCCAGAGGAGAGA-3
PAI1-F (for cloning)	5'-CAGGATGCAGATGTCTCCAG-3
PAI1-R (for cloning)	5'-GGTCAGGGTTCCATCACTTG-3'
HPRT-F	5'-CTTTGCTGACCTGCTGGATT-3'
HPRT-R	5′-TCCCCTGTTGACTGGTCATT-3'
uPA-F	5′-GGAGATGAAGTTTGAGGTGGAA-3′
uPA-R	5′-CTCCTTGGAACGGATCTTCAG-3′
uPAR-F	5′-TTGAAGATCACCAGCCTTACC-3′
uPAR-R	5′-GGTAACGGCTTCGGGAATAG-3′
PAI1-F	5′-CTGGTGAATGCCCTCTACTTC-3′
PAI1-R	5′-TGCTGCCGTCTGATTTGT-3′
